# Structure of astrotactin-2: a conserved vertebrate-specific and perforin-like membrane protein involved in neuronal development

**DOI:** 10.1098/rsob.160053

**Published:** 2016-05-04

**Authors:** Tao Ni, Karl Harlos, Robert Gilbert

**Affiliations:** Division of Structural Biology, Wellcome Trust Centre for Human Genetics, University of Oxford, Roosevelt Drive, Oxford OX3 7BN, UK

**Keywords:** astrotactin-2, membrane attack complex-perforin protein, X-ray crystallography, biophysical interaction analysis, neural migration control

## Abstract

The vertebrate-specific proteins astrotactin-1 and 2 (ASTN-1 and ASTN-2) are integral membrane perforin-like proteins known to play critical roles in neurodevelopment, while ASTN-2 has been linked to the planar cell polarity pathway in hair cells. Genetic variations associated with them are linked to a variety of neurodevelopmental disorders and other neurological pathologies, including an advanced onset of Alzheimer's disease. Here we present the structure of the majority endosomal region of ASTN-2, showing it to consist of a unique combination of polypeptide folds: a perforin-like domain, a minimal epidermal growth factor-like module, a unique form of fibronectin type III domain and an annexin-like domain. The perforin-like domain differs from that of other members of the membrane attack complex-perforin (MACPF) protein family in ways that suggest ASTN-2 does not form pores. Structural and biophysical data show that ASTN-2 (but not ASTN-1) binds inositol triphosphates, suggesting a mechanism for membrane recognition or secondary messenger regulation of its activity. The annexin-like domain is closest in fold to repeat three of human annexin V and similarly binds calcium, and yet shares no sequence homology with it. Overall, our structure provides the first atomic-resolution description of a MACPF protein involved in development, while highlighting distinctive features of ASTN-2 responsible for its activity.

## 1. Introduction

Perforin-like proteins (PLPs) have been identified in all forms of cellular life except, currently, Archaebacteria [1]. They represent a sub-branch of the largest known family of pore-forming proteins, the membrane attack complex-perforin/cholesterol-dependent cytolysin (MACPF/CDC) family [2,3]. The CDCs were identified in Gram-positive bacteria [4,5] and their mechanism of action has been thoroughly studied using a combination of structural and mechanistic approaches [6,7]. Perforins were separately identified, first as part of the complement membrane attack complex (MAC) [8,9] and then in the form of perforin-1 [10,11], which delivers granzymes from cytotoxic cells into target antigen presenting cells. The solution of the structures of complement C8α and a bacterial PLP revealed that MACPFs and CDCs belong to one homologous family of proteins [12,13], and that their identification as separate groupings was a historical accident [14].

The only basis for MACPF/CDC activity properly established so far is the oligomerization of many subunits in order to generate a pore-forming complex [15–19]. Binding to a target membrane serves the basic function of concentrating monomeric subunits on a planar substrate [14]. Then, concentration-dependent oligomerization occurs into a pre-pore complex before pore formation ensues [16,19–21]. It has been argued both that oligomerization to a complete ring of subunits is required for pore formation [7,22], and that incomplete rings (arcs) of MACPF/CDC subunits can insert into membranes to form pores, with the size of any individual pore-forming assembly being determined by the availability of further monomers for incorporation into the pre-pore [5,6,18,19,23,24]. Recently, it became clear that indeed individual types of MACPF/CDC protein can form functional pores using oligomers of variable size depending on precisely such a kinetically determined mechanism [18,19,25–28]. The same protein can generate functionally important lesions of different sizes in target membranes depending on prevailing conditions such as the concentration of protein and also, for example, pH [18,19,29,30].

As a subset of MACPF/CDC proteins, we define PLPs as being proteins which have been identified by sequence homology as sharing common evolutionary ancestry with perforin-1 [3,31]. These proteins are involved in a wide range of biological processes, including not only immunity (MAC, perforin-1 and perforin-2) [32–34] but also cell invasion and egress by apicomplexan parasites [35–37] and organismal development [38–43]. Our focus here is on a PLP especially identified as being involved in neurodevelopment, astrotacin-2 (ASTN-2) [39,40]. In addition to its role in neural migration control, a recent report has indicated that ASTN-2 is also part of the mechanism of planar cell polarity (PCP) determination in hair cells via the activation of non-canonical Wnt signalling Frizzled-6 receptors [44]. In this paper, we report structures representing a large portion of ASTN-2. These provide a blueprint for understanding its activity and also that of ASTN-1, which is also neurodevelopmentally significant [38], and to some extent the bone morphogenetic protein and retinoic acid inducible neural-specific proteins (BRINPs) [41,42].

ASTN-1 was first discovered because antibodies against it block neuron–glial interactions *in vitro* [38]; it was subsequently shown to be expressed in post-mitotic neuronal precursors of the cerebellum, hippocampus, cerebrum and olfactory bulb with a role in the establishment of laminar structures [45]. ASTN-1 is directly responsible for the formation of neuron–glial fibre contacts in the cerebellum [45–48], and the discovery of ASTN-2 as its intracellular counterpart showed how ASTN-1 contacts might be recycled through the endosomal system to enable the forward migration of neuronal cells [39] (electronic supplementary material, figure S1).

ASTN-2 is most highly expressed in the cerebellum but also in the hippocampus, cortex and olfactory bulb [39,49]. The expression of ASTN-1 and -2 is differentially regulated in terms of cellular location [39] and developmental stage [50]. While ASTN-1 is well expressed on the cell surface and in neurons forming glial fibre contacts via its C-terminal ectodomain, ASTN-2 is expressed there only very weakly (with ASTN-1 present, on 0.22% of cells tested; in the absence of ASTN-1, on 0.07% of cells tested) [39]. Instead, ASTN-2 localizes mostly to vesicles inside neural cells, giving rise to a punctate antibody staining pattern in the soma and along neuronal processes [39]. In agreement, co-expression of ASTN-1 and fluorescently labelled ASTN-2 in HEK293T cells shows them to be co-localized to a subset of RhoB^+^ endosomes (as well as in non-RhoB^+^ vesicles) [39]. As cerebellar granule neurons grow, ASTN-2 is found co-localized with clathrin at the base of the leading process and opposite an interstitial junction with the glial fibre, which suggests its identification with coated vesicles (electronic supplementary material, figure S1) [39]. Intracellular imaging reveals the cycling of vesicles bearing ASTN-1 from the anterior pole of the neuronal soma and the base of the leading process, into the cell, and down the leading process to form a new neuron–glial fibre junction towards its tip [39,40]. This has led to the hypothesis whereby ASTN-2 controls the recycling of ASTN-1-mediated contacts between the migrating neuron and glial fibre from the lagging to the leading edge of the moving cell [39] (electronic supplementary material, figure S1).

As might be expected given their role in neurodevelopment, data suggest that ASTN-1 and ASTN-2 are key to many aspects of basic mammalian neurobiology. For example, ASTN-1 is increased in rat brain following hippocampal injury, implying a role in repair processes [51], while ASTN-1 knockout mice display poorer balance and coordination than wild-type [47]. In humans, a comparative genomic hybridization study of a Russian cohort with intellectual disability identified an individual with a duplication of ASTN-1 showing multiple neurodevelopmental defects and delays as well as a number of non-neuronal phenotypic effects [52] which seem to map at least in part onto the PCP pathway [44,53].

Linkage of ASTN-2 to the development of the mammalian CNS is even stronger than that of ASTN-1. ASTN-2 has been implicated via genome-wide association studies in attention-deficit hyperactivity disorder (ADHD) [54], by copy number variant (CNV) analysis of a large human cohort in autism spectrum disorders (ASDs) [55] and in another CNV study in schizophrenia [56]. In one recent paper, a large set of neurodevelopmental disorder (NDD) subjects were compared with a set of population-based controls to identify 46 deletions and 12 duplications affecting ASTN-2; the NDD subjects demonstrated a variety of phenotypes including ASDs, ADHD, speech delay, anxiety and obsessive compulsive disorder [50]. In the same study, analysis of the spatio-temporal expression patterns of ASTN-1 and -2 in human brain samples from individuals of different ages showed ASTN-1 expression at consistently high levels, whereas ASTN-2 expression peaked in the early embryonic neocortex and postnatal cerebellar cortex [50]. Another recent paper identified a clinical link between ASTN-2-associated polymorphisms and the onset of Alzheimer's disease approximately 5 years earlier than the median [57].

**Figure 1.**
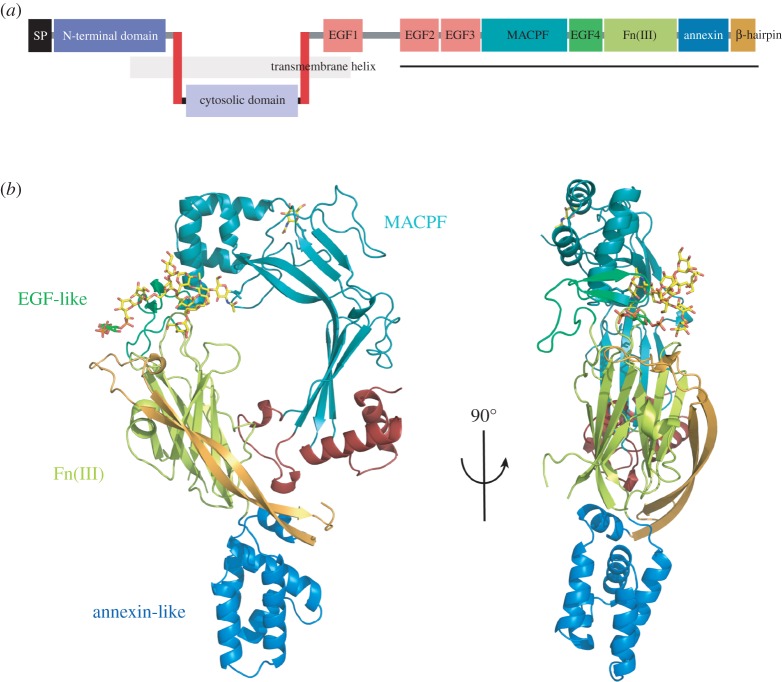
Overview of the structure of ASTN-2. (*a*) Schematic of the domain organization of ASTN-2 including its two transmembrane helices and the endodomain resolved here. (*b*) Two views of the ASTN-2 endodomain structure related by a 90° rotation, as shown. The different sub-domains (MACPF, EGF-like (EGF-4), Fn(III) and annexin-like) are highlighted and labelled directly; loop 1 and loop 2 are shown in red and the C-terminal β-hairpin packed back across the Fn(III) domain in yellow.

ASTN-1 and ASTN-2 have been shown to interact directly with each other *in vitro* in a calcium-independent way with multiple regions of each protein apparently contributing to their interface [39]. Both ASTN-1 and ASTN-2 are integral membrane proteins in which a large C-terminal domain (extracellular for ASTN-1, endosome luminal for ASTN-2; electronic supplementary material, figure S1*b*) is complemented by an N-terminal cytosolic domain suspended between two transmembrane α-helices [39] (figure 1*a*). The C-terminus of each protein is known to be detectable on the outside of cells in which they have been expressed and this defines their membrane orientation (electronic supplementary material, figure S1*b*) [39]. We suggest that just as the bulk of ASTN-1 projects outwards from the plasma membrane the bulk of ASTN-2 will project into the lumen of cellular endosomes, making it topologically equivalent to ASTN-2 with two transmembrane α-helices providing a firm anchor in the membrane bilayer (figure 1*a*; electronic supplementary material, figure S1*b*). Thus, the endosomal regions of ASTN-2 comprise a smaller N-terminal domain and a larger C-terminal domain whose structure we report here and which we refer to as its *endodomain*. The ASTN-2 endodomain is exactly equivalent to the cell surface-exposed ectodomain of ASTN-1; and it is the first in a class of integral membrane PLPs (which includes perforin-2) to have its structure determined, though perforin-2 differs from ASTN-2 (and ASTN-1) in having only a single transmembrane helix [33,58].

In describing here the structure of the endodomain of ASTN-2, we provide a framework for understanding its role in cell migration and tissue development. We describe its unique combination of a canonical MACPF/CDC domain with a minimal epidermal growth factor (EGF)-like repeat (EGF-4), a previously unobserved type of fibronectin type III (Fn(III)) domain in which an additional two β-strands are folded across the core, and an annexin-like domain. We have determined structures of the endodomain at pH 7.5 and pH 5 and of the MACPF/CDC domain alone at pH 4, and find the regions of ASTN-2 resolved to be remarkably insensitive to acidification. We have shown that the ASTN-2 endodomain binds inositol phosphates at a binding pocket located between the EGF-4 and Fn(III) domains and that the annexin-like domain binds calcium, as might be expected. ASTN-2 appears to be unique to vertebrates and its polypeptide sequence is extremely conserved, with 53% identity between jawless fishes and humans (last common ancestor, 485 Ma [59]) (electronic supplementary material, figure S2). Sequences are much more diverse among fish species than among terrestrial vertebrates, as befits their extreme antiquity as a group; in fact the sequences of land-dwelling vertebrates are remarkably constrained. It is tempting to suggest that the distinctive challenges of terrestrial life, and the fundamental role apparently played by astrotactin molecules in vertebrate nervous systems, has particularly constrained diversity on land. In any case, the isolation of astrotactins to vertebrates suggests they do play a key role specifically in the development of the vertebrate CNS, which provided the basis for the evolution of mammals leading, ultimately, to humans.

## 2. Results and discussion

### 2.1. Overall description of the ASTN-2 endodomain structure

We expressed and purified an ASTN-2 construct running from its second EGF-like repeat to the C-terminus (underlined in figure 1*a*) using HEK293 cells. Transient DNA transfection with the pHLsec vector previously described [60] enabled secretion into the cell medium, whence the protein was purified (see Material and methods for a full description of protein expression and purification). Figure 1*b* reports the overall structure of the ASTN-2 endodomain at pH 7.5 and comprising the MACPF/CDC, EGF-4, Fn(III) and annexin-like domains. The structure was solved using single isomorphous replacement with anomalous scattering (SIRAS) with a platinum-soaked derivative (see Material and methods for structure determination; see table 1 for data collection and refinement statistics). Although we verified that the expressed protein had remained intact such that EGF repeats 2 and 3 (EGF-2 and EGF-3) were present in the crystallized protein (electronic supplementary material, figure S3), they were not resolved in the structure, presumably due to disorder, though small-angle X-ray scattering (SAXS) data indicate that the domains are folded (see below and electronic supplementary material, figure S8*c*). The presence of EGF-2 and EGF-3 in the protein that crystallized combined with their invisibility in the crystal structure indicates that they are not important in the folding of the regions of ASTN-2 we do resolve. This is supported by normal modes analysis of the ASTN-2 endodomain which suggests that it constitutes a remarkably tightly folded unit, and by the equivalent interaction of ASTN-2 with inositol phosphates with and without these domains (see below and electronic supplementary material, figure S10). In normal modes analysis, the first six modes are rigid-body translational and rotational modes in three dimensions, so the first non-trivial mode is mode 7. Normal modes 7–11 indicate only breathing motions within the endodomain, and highlight the combination of the MACPF and Fn(III) as a particularly robust region of the structure (electronic supplementary material, figure S4*a*). We also solved the structure of the endodomain at pH 5 and of the MACPF/CDC domain alone at pH 4 (electronic supplementary material, figure S4*b*); these structures are compared with the neutral pH endodomain structure (figure 1*b*) below.

**Table 1.** Structure of the endodomain of astrotactin-2. Statistics for the highest resolution shell are shown in parentheses.

data collection	ASTN-2_601-1288_ (native)	ASTN-2_601-1288_ (Pt-derivative)	ASTN-2_649-984_	ASTN-2_649-1288_
space group	P1	P1 (Pt soak)	C121	P4_1_2_1_2
cell dimensions				
* a*, *b*, *c* (Å)	101.24, 108.19, 111.36	101.59, 106.93, 112.65	98.7,86.6, 108.36	103.9,103.9, 304.2
* α*, *β*, *γ* (°)	87.18, 84.5, 65.67	87.29, 84.68, 65.66	90, 111.74, 90	90, 90, 90
resolution (Å)	98.58–3.16	112.16–5.0	60.77–3.63	85.8–5.22
*R*_merge_	0.093 (0.890)	0.209 (1.111)	0.188 (1.547)	0.12 (3.047)
*R*_pim_	0.036 (0.602)	0.046 (0.323)	0.026 (0.705)	0.027 (0.643)
*I*/*σI*	17.1(1.4)	17.1 (5.5)	19.3 (1.2)	17.3 (2.0)
completeness (%)	99.9(99.3)	99.9 (99.8)	99.4 (98.4)	100 (100)
redundancy	8.5 (3.4)	26.3 (25.5)	49.3 (6.8)	23.8 (21.4)
CC_half	0.998 (0.433)	0.969 (0.936)	0.999 (0.517)	0.999 (0.798)

refinement	ASTN-2_601-1288_ (native)		ASTN-2_649-984_ pH 4	ASTN-2_649-1288_ pH 5
space group	P1		C121	P4_1_2_1_2
resolution (Å)	98.58–3.16		60.77–3.63	85.8–5.22
no. reflections	627 041 (18 219)		475 699 (4647)	164 862 (10 473)
unique reflections	73 362 (5382)		9653 (688)	6915 (490)
*R*_work_/*R*_free_ (%)	23.3 (25.9)		27.7 (29.9)	32.8 (35.9)
no. atoms				
protein	17 842		4034	4456
ligand/ion	568		56	154
average *B*-factors				
protein	135.9		196	335
ligand/ion	224.1		217	348
RMSDs				
bond length (Å)	0.008		0.003	0.005
bond angles (°)	1.18		0.67	0.85

### 2.2. MACPF and EGF-like domain structures

**Figure 2.**
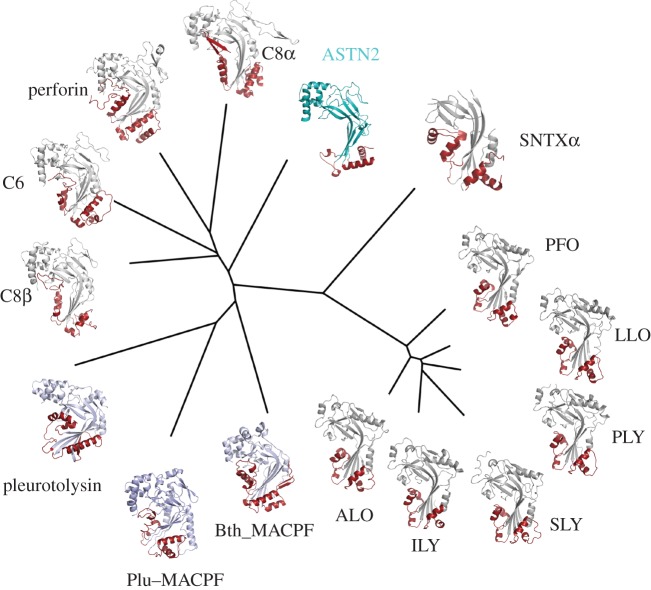
Structural phylogeny of known MACPF domain structures. The ASTN-2 MACPF domain core bent sheet is coloured cyan, with loop 1 and loop 2 in red; in all other cases the core sheet is coloured grey. From ASTN-2 clockwise the proteins shown are as follows: stonustoxin (SNTXα) from stonefish (PDB ID 4WVM); perfringolysin (PFO) from *Clostridium perfringens* (1PFO); listeriolysin (LLO) from *Listeria monocytogenes* (4CDB); pneumolysin (PLY) from *Streptococcus pneumoniae* (4QQA); suilysin (SLY) from *Streptococcus suis* (3HVN); intermedilysin (ILY) from *Streptococcus intermedius* (1S3R); anthrolysin (ALO) from *Bacillus anthracis* (3CQF); *Bacteriodes thetaiotamicron* MACPF (Bth_MACPF) protein (3KK7); *Photorhabdus luminescens* MACPF (Plu_MACPF) protein (2QP2); pleurotolysin from fungus *Pleurotus osteatus*; complement C8β from human (3OJY); complement C6 from human (3T5O); perforin from mouse (3NSJ) and complement C8α from human (3OJY).

The N-terminus of the endodomain structure is formed by the canonical MACPF domain consisting of a central, broken, antiparallel four-stranded β-sheet that possesses a distinctive central approximately 90° bend. Figure 2 shows a structure-based phylogenetic comparison of the ASTN-2 MACPF domain and other known equivalent structures from the MACPF/CDC superfamily. The most distinctive features of MACPF/CDC domains are two loops containing α-helices which link the core domain β-strands and which undergo refolding in pore-forming family members to generate a partial or complete β-barrel inserted in the membrane [16,61–63] (figure 2). In ASTN-2, the first such loop (loop 1, linking strands β1–β3 and β4–β5), comprises 19 residues and contains a single-turn 3_10_-helix (η1) and which packs against the Fn(III) domain interface (see below; see electronic supplementary material, figure S5 for sequence alignment and secondary structure). The equivalent region in perforin-1 contains 55 residues and two substantial α-helices; and in perfringolysin (and all CDCs) approximately 33 residues and three short multi-turn helices (electronic supplementary material, figure S6*a*). The loop 1 region of the ASTN-2 structure is therefore an outlier with respect to MACPF/CDC proteins as a whole, due to its brevity. The second loop (loop 2; linking strands β6–β7 and β8–β9) consists of 54 residues and contains two substantial α-helices (α3 and α4). Loop 2 is solvent exposed in contrast with the packing of loop 1 against the Fn(III) domain. The equivalent region to ASTN-2 loop 2 in perforin-1 contains 59 residues (four α-helices, two short, two long) and in perfringolysin 30 residues (three helices). Thus, while loop 1 of the ASTN-2 MACPF/CDC domain is unusually short, loop 2 is similar in length to that of perforin-1 and quite a bit longer than that of the CDCs. Most critically, however, the two MACPF domain loops are mismatched in length.

All MACPF/CDC proteins that form pores have to date been shown to do so using a β-stranded barrel in which each subunit contributes two β-strand hairpins to generate a structure similar to a bacterial outer membrane porin, but formed by multiple subunits rather than a single polypeptide chain [3,61]. This requires the two β-hairpins to be not significantly different in length to each other and that they are long enough to span a bilayer membrane. Two factors suggest that ASTN-2 loops 1 and 2 may not function in pore formation or that, if they do, the mechanism of pore formation by ASTN-2 differs significantly from that of the pore-forming members of the MACPF/CDC superfamily (e.g. perforin-1, C8α, C6, pleurotolysin, perfringolysin, etc.; figure 2).

Firstly, the shortened ASTN-2 loop 1, packed against the Fn(III) repeat, is too short to span a bilayer within a β-barrel structure such as the CDCs, perforin and some PLPs are known to form [16,17,61,63,64]. Furthermore, while both perforin-1 and perfringolysin show an amphipathic sequence pattern in their respective loops 1 and 2, in ASTN-2 this pattern is lost (electronic supplementary material, figure S6*a*). This is most striking in loop 1, but even loop 2 is more hydrophobic than the equivalent regions of perforin-1 and perfringolysin, and rather than a hydrophobic–hydrophilic pattern alternating every residue, the alteration seems to be more two-by-two. If loop 2 inserts into a membrane to form a pore this suggests it could do so as a helical structure rather than in the form of a β-hairpin—but whether ASTN-2 really forms membrane pores is unknown.

Secondly, while the exposed, unabbreviated ASTN-2 loop 2 is similar in length to the equivalent region of perforin-1, its large mismatch in length with loop 1 (ASTN-2 loop 1 is more than 30 residues shorter than loop 2) means that they could not pair in β-barrel formation. Yet for each other member of the MACPF/CDC superfamily, the two equivalent loops are similar in length to each other; this mismatch also suggests that ASTN-2 may not be a pore-forming protein, at least not in the way other MACPF/CD protein family members are.

The MACPF domain is completed by a single-turn 3_10_ helical turn (η2) and a final α-helix (α5; four turns) which are paired structurally with α1 and α2 to form a head region from which the MACPF domain β-strands extend. This head is linked to the Fn(III) domain by the EGF-4 domain module, which contains β10 and β11 and matches best to the integrin β_3_-subunit PSI domain EGF-like folds according to the SCOP database (family g.3.11.6) (electronic supplementary material, figure S6*b*); the two disulfide bridges in the EGF-4 domain superpose perfectly with those in the integrin β_3_ subunit PSI domain [65]. EGF repeats are also found acting as linker domains in perforin-1 itself and in complement C8α and C8β, at points which constitute hinges during the pre-pore to pore transition. To provide for comparison of the EGF repeats found in perforin, other MACPF proteins and other proteins identified as having similarly structured regions to the ASTN-2 EGF-4 repeat, we performed a structural phylogenetic analysis (electronic supplementary material, figure S6*b*). The EGF-like repeats compared were those from perforin-1, C8α and C8β; the PSI domain of integrin subunit β_3_; and an EGF repeat from P-selectin as well as EGF-4 from ASTN-2. As shown, it seems that the EGF repeat found in perforin-1 itself and EGF-4 in ASTN-2 share a common line of ancestry, separate from the EGF repeats found in other immune system MACPFs (C8α and C8β). ASTN-2 also seems to be closer to the common point of origin of itself and perforin-1 and thus perhaps to represent a more ancient structural form; indeed the perforin-1 EGF repeat is rather an extreme structure compared with ASTN-2 EGF-4 and the EGF repeats from C8α and C8β.

The junction between the EGF-4 and Fn(III) domains contains density which is not part of the ASTN-2 polypeptide and which can plausibly be modelled as an inositol triphosphate bound within a surface cavity. The binding mode of this species and mutational and binding data which support its identification with a phosphorylated inositol are described below.

### 2.3. Structures at pH 5 and pH 4

The movement of ASTN-2 through the endolysosomal system suggests that its activity might be regulated by pH. In an attempt to investigate the effect of reduced pH on the ASTN-2 structure, we obtained crystals of constructs consisting of EGF-3 to the C-terminus (residues 649–1288) at pH 5 and of EGF-3 plus the MACPF domain at pH 4 (residues 649–984), though in neither case was EGF-3 resolved in the resulting structures. The conformations of the lower pH forms of ASTN-2 are very similar to the equivalent regions of the neutral pH structure (electronic supplementary material, figure S4*b,c*) (RMSDs 0.23 Å (pH 5) and 1.52 Å (pH 4)). The region of greatest variation at pH 4 is loop 1 between β3 and β4, where 3_10_ helix η1 shifts approximately 2 Å towards the Fn(III) domain on acidification. Yet overall these regions of ASTN-2 seem remarkably insensitive to pH suggesting that, if pH is a triggering factor in ASTN-2 activity as it moves through the endolysosomal system, the sensor is not in these parts of the structure.

### 2.4. Fibronectin type III domain structure

The Fn(III) domain (β12–β15, π1, β16–β19 with the start of β16 and the end of β19 tied together by a disulfide bond) follows directly on from EGF-4 and has the unexpected feature that it is complemented by a 54-residue β-hairpin from the ASTN-2 C-terminus (β20–β21) that folds across the Fn(III) β-sandwich, clasping the edges of both sheets. As such it seems to lock the C-terminus back to the centre of the endodomain, rigidifying the structure (electronic supplementary material, figure S4*a*). The loop between the hairpin strands projects towards EGF-4 and towards the inositol phosphate binding site, suggesting that this point in the structure may be a key point in its conformational regulation.

The MACPF domain contains two glycosylation sites; one at the top of the domain (N719) was trimmed back by EndoF1 (see Material and methods) to a single *N*-acetyl galactosamine residue. The other (N732) is occupied by a high-mannose glycan sidechain protected from glucosidase trimming that appears to assist in the stabilization of the MACPF/EGF-4/Fn(III) domains cassette (figure 1*b*; electronic supplementary material, figure S7). The principal contacts made by this sugar sidechain with EGF-4 and the Fn(III) surfaces are formed by the two *N*-acetyl galactosamine moieties and terminal mannoses of the glycan sidechain branches. The role of the glycan sidechain in stabilizing the MACPF/EGF-4/Fn(III) domains cassette is indicated by the effects of a N > Q mutation knocking out its sequon. Such a construct is not secreted during mammalian cell expression, suggesting it is misfolded.

### 2.5. Details of the interface between the MACPF and Fn(III) domains

**Figure 3.**
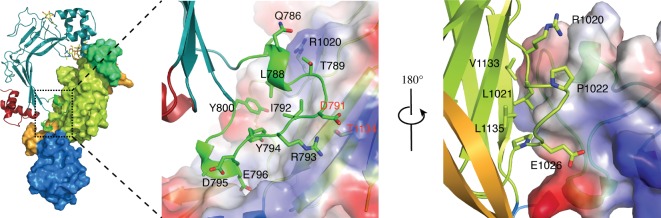
Close-up of the MACPF domain–Fn(III) domain interface. On the *left* is an overview in which the MACPF domain is shown in ribbon format and the EGF-4, Fn(III) and annexin-like domains with rendered surfaces. The domains are coloured as in figure 1. In the *centre* is a close-up view of the MACPF domain–Fn(III) interface in the same orientation with the Fn(III) surface rendered by charge from −5 kT/e (red) to +5 (blue). On the *right* is an equivalent view but rotated by 180° as shown, bringing the C-terminal β-hairpin packing across the Fn(III) domain into view.

The interface between the MACPF and Fn(III) domains is formed by the truncated loop 1 between strands β3 and β4 of the MACPF domain (figure 1*b*). The residues involved in the interactions are mostly charged (e.g. Arg793, Asp791, Glu796) though Phe800 projects towards the domain interface (figure 3). Despite the involvement of charged sidechains there do not seem to be direct electrostatic contacts formed between the domain surfaces, rather the presenting faces of the MACPF and the Fn(III) domains have a shape complementarity (figures 1*b* and 3). This suggests the possibility of breathing movements (supported by normal modes 8 and 9; electronic supplementary material, figure S4*a*) and even of a significant conformational change caused by movement of the MACPF away from the Fn(III) domain which might be enabled if the C-terminal β-hairpin ungrasps the Fn(III) domain. In an attempt to probe the functional significance of the MACPF/Fn(III) interface, we introduced a disulfide bond to lock the two together (Asp791Cys and Thr1134Cys mutations); this resulted in a form of the protein which was secreted poorly from HEK293T cells, although SAXS data suggest that the protein that is secreted is folded like wild-type ASTN-2 (electronic supplementary material, figure S8).

Structural phylogenetic analysis of the Fn(III) domain excluding the long C-terminal β-hairpin (β20–β21) indicates that this domain is closest in structure to Fn(III) repeat 8 from human fibronectin itself, and occupies a single branch with this domain and a Fn(III) domain from tenascin (electronic supplementary material, figure S6*c*). It is certainly therefore a bona fide Fn(III) domain despite its unique complementation with the β20–β21 hairpin, for which there are no currently described structural parallels.

### 2.6. The annexin-like domain

**Figure 4.**
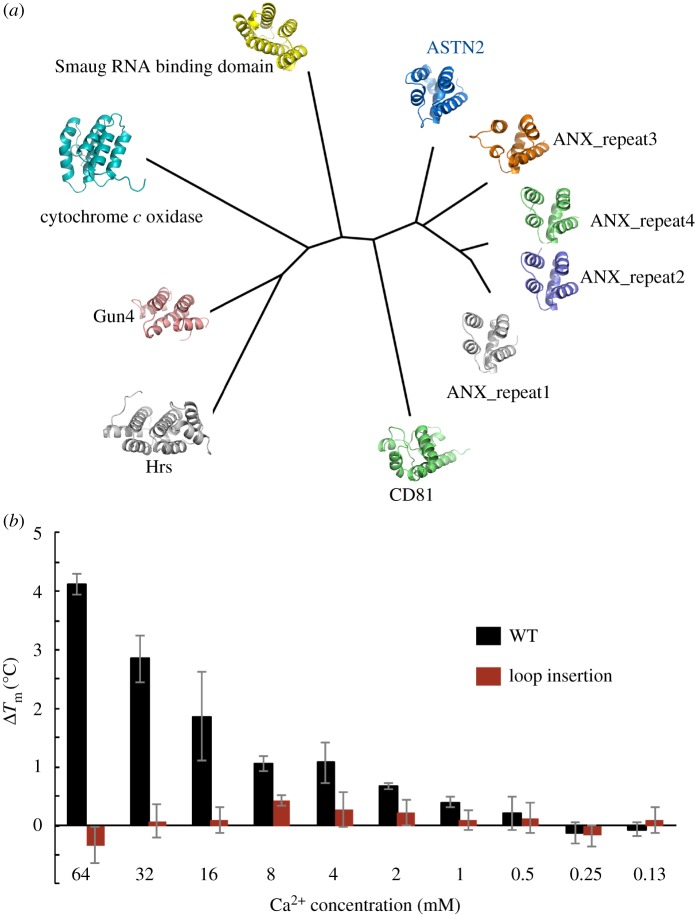
Annexin-like domain homology and calcium binding. (*a*) A structural phylogeny, constructed as described in the Material and methods, comparing the annexin-like domain of ASTN-2 with close structural homologues. Clockwise from the ASTN-2 domain as labelled, the structures shown are the repeats 3, 4, 2 and 1 from human annexin V (ANX, PDB ID 1AVH); the extracellular domain from human CD81 (1G8Q); a domain from Hrs1 from *Drosophila melanogaster* (1DVP); from Gun4 of *Thermosynechococcus elongates* (1Z3X); from bovine cytochrome *c* oxidase (1OCR); and from Smaug RNA-binding protein from *Drosophila melanogaster* (1OXJ). (*b*) Melting temperature shifts for the ASTN2_601-1288_, both as wild-type (WT) sequence and with the calcium-binding motif disrupted (loop inserted). Experiments were performed in triplicate and the mean ± s.d. is reported.

The annexin-like domain is positioned between the Fn(III) domain and the long β-hairpin folded across it (β20–β21) but, functionally, forms the tip of the ASTN-2 endodomain. An annexin-like domain is an unexpected feature of the ASTN-2 structure (α6–α11) which despite undetectable sequence homology with annexin itself superimposes very well on an annexin single repeat (figure 4*a*; see electronic supplementary material, figure S9 for a superimposition). Annexin itself consists of four tandem repeats of a common fold [66] and it is striking that the ASTN-2 annexin-like domain is closer in structure to human annexin repeat 3 (RMSD 1.45 Å) than human annexin repeat 3 is to repeat 1 (RMSD 1.58 Å). Thus, despite no sequence homology ASTN-2 and human annexin have very similar structures; their charge distributions, however, are very different (electronic supplementary material, figure S9*b*). For example, with the annexin V domains and the ASTN-2 annexin-like domain all oriented equivalently, the most-similar annexin V domain 3 presents a highly positively charged face whereas ASTN-2 presents a mixed face with regions of positive and negative charge.

Like annexin itself, the ASTN-2 annexin-like domain binds calcium, resulting in a stabilization of its structure as evidenced by an increased temperature of melting (figure 4*b*). This effect is confirmed by mutation of the putative calcium-binding residues within the annexin-like domain, which knock out the stabilizing effect of the presence of calcium (figure 4*b*). Annexin-like domains are known for their capacity to remodel membranes, triggered by calcium binding, and have also been suggested to be involved in the formation of pores in membranes [67]—both are possible biological roles of the ASTN-2 annexin-like domain. The MACPF/EGF-4/Fn(III) domains cassette is further stabilized by a disulfide bridge that caps the whole annexin-like fold, covalently linking its start (a linker region just before α6) to its end (a linker region in α11 which leads into β20).

### 2.7. Inositol phosphate binding site

**Figure 5.**
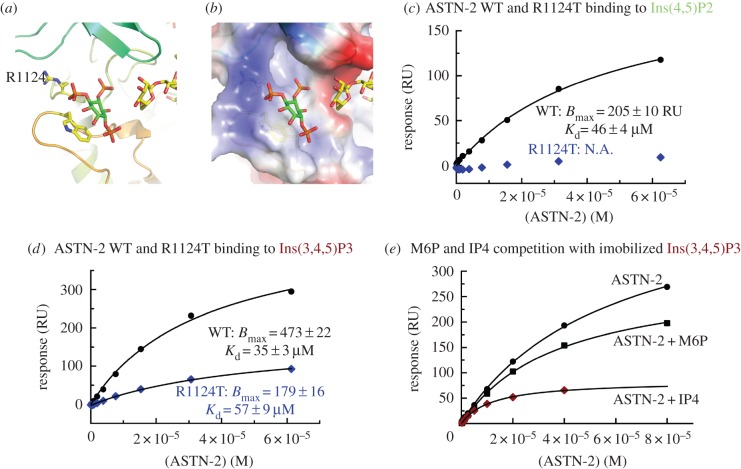
Inositol phosphate binding by ASTN-2. (*a*) Close-up of the inositol triphosphate modelled into extra density, one phosphate in electrostatic interaction with arginine 1124, a position occupied by a threonine in ASTN-1 and the inositol ring π-stacked with tryptophan 1259. (*b*) Surface charge representation of the inositol triphosphate binding pocket. (*c*) Wild-type (WT) ASTN-2 and an R1124T mutant interacting with immobilized inositol diphosphates, as measured by surface plasmon resonance (SPR). (*d*) Wild-type ASTN-2 and an R1124T mutant binding to immobilized inositol triphosphate, again measured by SPR. (*e*) A competition assay for the binding of ASTN-2 to the triphosphorylated inositide alone and with competition from Ins(1,3,4,5)P_4_ (IP4) and mannose-6-phosphate (M6P) in solution. The affinity of ASTN-2 for the immobilized Ins(3,4,5)P_3_ is much less affected by the presence of mannose-6-phosphate in solution than by the presence of free Ins(1,3,4,5)P_4_.

As mentioned above, the crystal structure contains density consistent with an inositol triphosphate species being bound in a shallow pocket formed by loops from EGF-4 and the β-hairpin slung across the Fn(III) domain. Initially, we identified difference density of unknown origin in this area which could be modelled as an inositol triphosphate, but faced a need to demonstrate its identity. We were assisted here by the observation that although ASTN-2 interacts with Ins(3,4,5)P_3_ and Ins(4,5)P_2_ in a surface plasmon resonance (SPR)-based experiment (*K*_d_ = 30 µM) (figure 5 and electronic supplementary material, figure S10*a*), ASTN-1 does not (electronic supplementary material, figure S10*b*). A comparison of the sequences of ASTN-2 and ASTN-1 in this region indicated that an arginine apparently interacting with Ins(3,4,5)P_3_ in the ASTN-2 crystal structure is replaced by a threonine in ASTN-1. Mutation of ASTN-2 Arg1124 to Thr resulted in a form of the protein which, like ASTN-1, did not bind Ins(4,5)P_2_ and had much-reduced affinity for Ins(3,4,5)P_3_ (figure 5). It is possible that the binding of inositol phosphates is non-specific, for example that ASTN-2 binds specifically to mannose-6-phosphate instead. We therefore performed a competition assay (figure 5*c* and electronic supplementary material, figure S10*c*) to demonstrate that ASTN-2 makes a specific choice of Ins(3,4,5)P_3_ over mannose-6-phosphate in our hands. We do not, however, know the biological significance of this interaction—which could be either that ASTN-2 binds phosphorylated phosphatidylinositol lipid species, or that it binds to inositol phosphate second messengers, or to another phosphorylated or similarly modified carbon ring system. The location of the binding pocket would permit either or both kinds of phosphorylated inositol species to be bound, though we are not aware of free inositol phosphates being found in either the extracellular or endosomal compartments.

## 3. Conclusion

Here we have described the structure of a major portion of ASTN-2, the first structure from an integral membrane MACPF/CDC domain-containing protein. ASTN-2 is closely related to not only ASTN-1 but also the BRINP proteins which, similarly, function in neural cell migration and guidance. Other integral membrane MACPF/CDC proteins include perforin-2, a macrophage-specific form of perforin involved in bacterial killing [33,58], and torso-like, which plays a role in early development of the *Drosophila* embryo [43,68]. The structure we describe is of relevance for understanding ways in which both closely related proteins (ASTN-1 and the BRINPs) and also those more distantly related might function biologically. For example, we have shown that the MACPF/CDC domain of ASTN-2 does not equip it to form pores by a mechanism similar to that of perforin-1 and the complement MAC, or bacterial members of the family [3]. We have also shown how additional domains associated with the MACPF/CDC homologous region of ASTN-2 contribute to its structure and the basis on which it might act, by binding calcium (the annexin-like domain) and phosphorylated inositol species. Our structure reveals how different functions, such as membrane binding via an annexin-like domain, have been co-opted to enable the functioning of a modified MACPF/CDC domain in a novel context, as well as currently uniquely described mechanisms for bracing a protein structure by the folding of a long hairpin across the cleft of a Fn(III) domain. The stage is now set for further experiments investigating the details of ASTN-2 functioning in neuronal and other cells, including its role in PCP determination, and also for the determination of structures for full-length ASTN-2, for ASTN-1 and the BRINPs which, like ASTN-2, have modified MACPF/CDC domains seemingly lacking a basis on which to form pores such as other MACPF/CDC proteins form. Without an easy analogy to pore formation by perforin/MAC or the CDCs it is impossible to say why a MACPF/CDC domain is of advantage for the activity of ASTNs and BRINPs, but our structural studies provide an essential stepping stone along the way to an answer. One possibility is that ASTN-2 has a membrane fusion-type activity [14].

## 4. Material and methods

### 4.1. Design of constructs, cloning, mutagenesis and sequence analysis

The full-length cDNA clone of Human ASTN-2 (UniprotKB/Swiss-prot O75129, isoform 2) was obtained from Source Bioscience (UK) and the expression constructs reported here are all based on the pHLsec vector [60]. For production of the larger portion of ASTN-2′s C-terminal endodomain (residues 601–1288), the construct was cloned in-frame with a Human Rhinovirus (HRV)-3C protease cleavage site, followed by a monoVenus and 8xhis tag. Other constructs (residues 701–1288 and residues 701–984) used in this study were all also cloned in-frame with hexahistidine. Site-directed mutagenesis was introduced by overlapping PCR [69]. Construct sequences were verified by DNA sequencing (Source Bioscience). Sequence alignments were performed using Clustal Omega and displayed using Espript 3 [70] (electronic supplementary material, figure S5). Conservation of sequence was calculated using ConSurf [71] for plotting on the surface of the ASTN-2 structure (electronic supplementary material, figure S9*c*).

### 4.2. Protein expression and purification

The expression constructs were transiently transfected into HEK293T cells using polyethylenimine (PEI) as described before [60]. All the recombinant proteins used in this study were expressed, secreted from HEK293T cells and purified from the media. For crystallization, the proteins were produced in the presence of 5 µM of the class I α-mannosidase inhibitor, kifunensin, explaining the mannose-rich (unedited) glycan [72]. For all other experiments, the proteins were produced without kifunensin.

The media containing the secreted recombinant proteins were harvested 5–6 days after transfection, then filtered through 0.22 µm filters and dialysed against phosphate-buffered saline (pH 7.4) overnight before loading onto a pre-equilibrated HisTrap HP column (GE Healthcare, 5 ml) in a cold room. The column with proteins bound was washed with 10 column volumes of Tris buffer (20 mM Tris–HCl pH 7.5 500 mM NaCl) before being eluted with 10–500 mM imidazole with a gradient concentration in the same Tris buffer. GST-3C was added into the eluates to remove the C-terminal monoVenus-8xhis where necessary. The glycan chains of proteins for crystallization were trimmed using Endo-F1. Subsequently, the eluates were buffer-exchanged into low salt buffer (20 mM Tris–HCl, pH thinsp;8.5, 50 mM NaCl) and loaded onto a pre-equilibrated Q column (GE Healthcare, 5 ml) and eluted with the same buffer containing a higher concentration of NaCl. This step efficiently removed albumin contamination. Finally, proteins eluted from the Q column were applied to a size-exclusion chromatography column (Superdex 200 16/600, GE Healthcare) and homogeneous proteins were pooled together, concentrated to about 10 mg ml^−1^, flash-frozen in liquid nitrogen and stored at −80°C until further use.

### 4.3. Crystallization, data collection and processing

Crystallization screening was carried out using commercially available crystallization reagents by vapour diffusion methods in sitting drop format in 96-well plates. For crystallization of ASTN2_601-1288_, 200 nl sitting drops of protein solutions (12 mg ml^−1^ in 10 mM HEPES pH 7.5, 150 mM NaCl) were mixed with 100 nl of precipitant (12% PEG 20000, 150 mM KSCN, 0.1 M Bis-tris propane pH 7.5–8.5) and equilibrated against 90 µl of crystallization condition. The small needle-like crystals usually appeared after a week and microseeding with the needle-like crystals yielded reasonably-sized crystals [73]. For crystallization of ASTN-2_649-984_, the protein was concentrated to 15 mg ml^−1^ and crystallized in 0.1 M citric buffer, pH 4.0–4.5, 20% PEG 1000. ASTN-2_649-1288_ was crystallized in 0.1 M MES, pH 5.0–6.0, 5% PEG 6000. Platinum derivative crystals of ASTN2_601-1288_ were prepared by soaking the crystals with concentrated PtCl_4_ for 1 h; 25% (vol/vol) glycerol prepared in mother liquor was used as cryoprotectant for both native and platinum-soaked crystals, which were subsequently flash-frozen in liquid nitrogen. Diffraction data were collected at 100 K at Diamond Light Source (Didcot, UK) on beamlines I02, I03, I04, I04-1 and I24. Although most of the crystals diffracted poorly with a high-resolution limit up to 3.6 Å, a native dataset with higher resolution (3.16 Å) was eventually achieved by screening a number of crystals. An inverse-beam data collection strategy was employed to accurately record the anomalous signal from platinum derivative crystals. Diffraction data were indexed, integrated and scaled using the Xia2 pipeline [74].

### 4.4. Structure determination and refinement

The structure of ASTN-2 endodomain was solved by single isomorphous replacement with anomalous scattering (SIRAS). A high-redundancy platinum derivative dataset was achieved by combining 21 datasets selected from seven crystals. The Pt sites were initially identified using HKL2MAP [75] with SHELXC [76], which were subsequently fed into PHENIX.autosol [77]*.* The resolution cut-off was set to 6.5 Å and solvent content to 0.6 to obtain initial phases. Density modification with non-crystallographic symmetry (NCS) averaging and phase extension to the full resolution of the native dataset resulted in an interpretable map by RESOLVE [78]. A polyalanine model was initially built into the electron density map manually in Coot [79], followed by refinement in PHENIX.refine [77] with external (Pt-SAD) phase restraints. The electron density of amino acid residue side chains appeared after two cycles of model building and refinement. Further density modification in RESOLVE with NCS calculated from the polyalanine model resulted in an excellent electron density map, which could then be fed into PHENIX.autobuild. The model was completed by manual rebuilding in Coot and refinement in refmac5 and PHENIX. The crystal structure of the MACPF domain C-terminus at pH 5 and of the MACPF domain alone at pH 4 were solved by molecular replacement using copy A of the structure solved at pH 7.5 in PHASER and refinement was carried out in PHENIX [77]. Surface electrostatic potentials for ASTN-2 and other proteins were calculated using APBS [80]. The crystallographic statistics are listed in table 1 and all models were validated with Molprobity software [81]. Data collection and structure determination will be described in detail elsewhere.

### 4.5. Surface plasmon resonance

The SPR experiments were performed using a Biacore T200 machine (GE Healthcare Life Sciences) at 20°C in 10 mM HEPES, pH 7.5, 150 mM NaCl, PtdIns(3,5)P_2_ (C-35B6a), PtdIns(4,5)P_2_ (C-45B6a), PtdIns(3,4,5)P_3_ (C-39B6a) and Ins(1,3,4,5)P_4_ (Q-1345) were purchased from Echelon Biosciences. To immobilize the biotinylated inositol phosphate onto the sensor chip, a BIAcore CM5 chip (GE Healthcare Life Sciences) was first derivatized with streptavidin following the manufacturer's instructions, and inositol phosphates were then injected on to channels 2 and 4 of the biosensor surface, leaving channels 1 and 3 as empty controls. The analyte with twofold serial dilutions was applied at a flow rate of 20 μl min^−1^ for 180 s followed by 300 s of dissociation time. The biosensor chip was regenerated by 0.1% SDS after each running cycle. To perform the competition assay, the analyte (ASTN-2_701-1288_) was incubated with a 10-fold molar concentration of Ins(1,3,4,5)P_4_ and mannose-6-phosphate for 45 min, respectively, before being serially diluted in running buffer. The data were fit with the 1 : 1 Langmuir adsorption model (*B* = *B*_max_*C*/(*K*_d_ + *C*), where *B* is the response of bound analyte and *C* is the concentration of the analyte in the sample) to calculate the dissociation constant (*K*_d_) using BIAcore BIAanalysis software.

### 4.6. Thermofluor

Thermofluor experiments were conducted using a real-time PCR machine [82]. Identical protein samples (4 µg) with SYPRO orange stain (Life Technologies S-6650, final concentration 3×) were mixed with serial concentrations of CaCl_2_ (0.13–64 µM) in 10 mM HEPES, pH 7.5, 150 mM NaCl. Fluorescence measurements were recorded from 298 to 372 K with a 1 K temperature increase each cycle. The melting temperature *T*_m_ (the midpoint of the unfolding transition) was calculated from the melting curve.

### 4.7. Small-angle X-ray scattering

SAXS data were collected on B21 at the Diamond Light Source (Didcot, UK). The measurements were carried out at 293 K in 10 mM HEPES, pH 7.5, 150 mM NaCl buffer with a momentum transfer range of 0.004 Å^−1^ < *q* < 0.45 Å^−1^, where q = 4*π*sin(*θ*)/*λ* and 2*θ* is the scattering angle. SAXS datasets for WT ASTN-2 were collected at four concentrations: 4.92 mg ml^−1^, 2.49 mg ml^−1^, 1.27 mg ml^−1^ and 1 mg ml^−1^, and the disulfide bond locking mutant was measured at 1 mg ml^−1^. Eighteen frames of measurements were recorded and the frames without radiation damage were averaged. The scattering intensity from buffer alone was subtracted from the averaged data to obtain the protein scattering in solution. Data reduction was carried out using the ATSAS package [83]. For wild-type ASTN-2, a merged dataset was obtained by combining the low-angle part of the low-concentration dataset with the high-angle part of the high-concentration dataset. The radius of gyration (*R*_g_) was calculated from a Guinier plot using AutoRg [83]. Particle distance distribution function *P*(*r*) was calculated in GNOM [84] using the low-resolution range of the dataset (0.01 Å^−1^ < *q* < 0.15 Å^−1^). An *ab initio* model was then calculated from the *P*(*r*) function using dammif and dammin [85]. EGF-2 and EGF-3 domain structures were predicted using Phyre2 [86] using tandem EGF domains of Del-1 as a threading model (PDB code: 4D90 [87]). The crystal structure of ASTN-2 was then fitted manually in PUMOL.

### 4.8. Analytical ultracentrifugation

Sedimentation velocity experiments were performed using a Beckman Optima XL-I analytical ultracentrifuge equipped with absorbance and interference optics. Double-sector 3 mm centrepieces were used with protein samples at 1 mg ml^−1^ and using absorbance measurements at 280 nm. Experiments were performed at 20°C, taking sample distribution scans every 6 min. Data were analysed using SEDFIT software using the *c*(*s*,*f*/*f*_0_) method of interpretation to generate sample distributions in *s* (sedimentation coefficient) without assuming a particular number of species [88]. The resulting distributions were curve fit in ProFit (QuantumSoft, Uetikon am See, CH).

### 4.9. Structural phylogeny calculation

Superimposition of homologous protein structures was performed using SHP [89] as previously reported [90]. The phylogenetic tree was calculated using a pairwise evolutionary distance matrix determined from the superimposed domains. The tree representation was generated using the programs FITCH and DRAWTREE as part of the PHYLIP package [91].

### 4.10. Normal modes analysis

Normal modes analysis was performed using the online server elNémo [92]. The vibrational movements of the structure were visualized by superimposition of models within each of the six lowest frequency non-trivial normal modes in Chimera [93].

## Supplementary Material

Supplementary Information

## Data accessibility

The structures reported in this paper have been deposited in the RCSB protein databank, PDB IDs 5J67 (pH 7.5), 5J68 (pH 5) and 5J69 (pH 4).
