# Supplementary material for: Structure of astrotactin-2: a conserved vertebrate-specific and perforin-like membrane protein involved in neuronal development
Source: Open Biol. 2016 May 4;6(5):160053. doi: 10.1098/rsob.160053 (PMC4892435; doi:10.1098/rsob.160053)
Supplement: Supplementary Information [file rsob160053supp1.pdf]

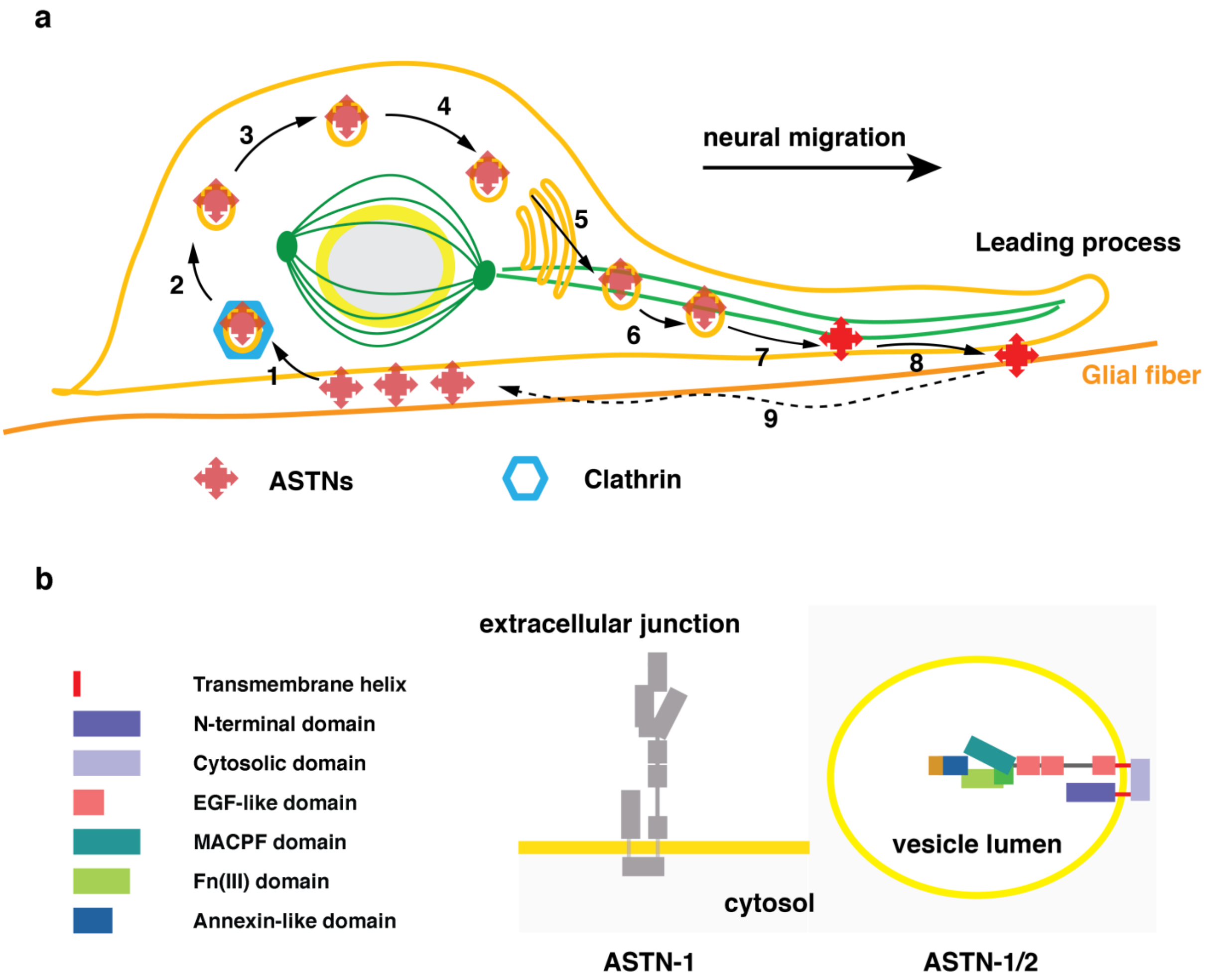

Supplementary Figure 1 | Schematic overview of ASTNs in neuronal migration. (a) Schematic of roles played by ASTNs 1 and 2. ASTN-1-mediated adhesions undergo endocytosis into clathrin-coated vesicles dependent on ASTN-2 (1); the vesicles cycle through early and recycling endosomes (2-4) and undergo microtubular migration (5-7) until the ASTN1 is re-deposited towards the leading process (7) to form a new adhesion (8) which will be recycled again (9) in step with the cell migration. **(b)** Outline of ASTNs domain architectures and intracellular localisation. Both ASTNs are integral membrane proteins, with two transmembrane helices projecting a large C-terminal domain into the extracellular junction (ASTN-1) or endosomal vesicle lumen (ASTN-1 and ASTN-2), while exposing cytosolic domains on the other side of membrane. Panel **a** is after Perrin Wilson et al., “Astn2, A Novel Member of the Astrotactin Gene Family, Regulates the Trafficking of ASTN1 during Glial-Guided Neuronal Migration”, *J. Neurosci.* **30**, 8529-8540 (2010).

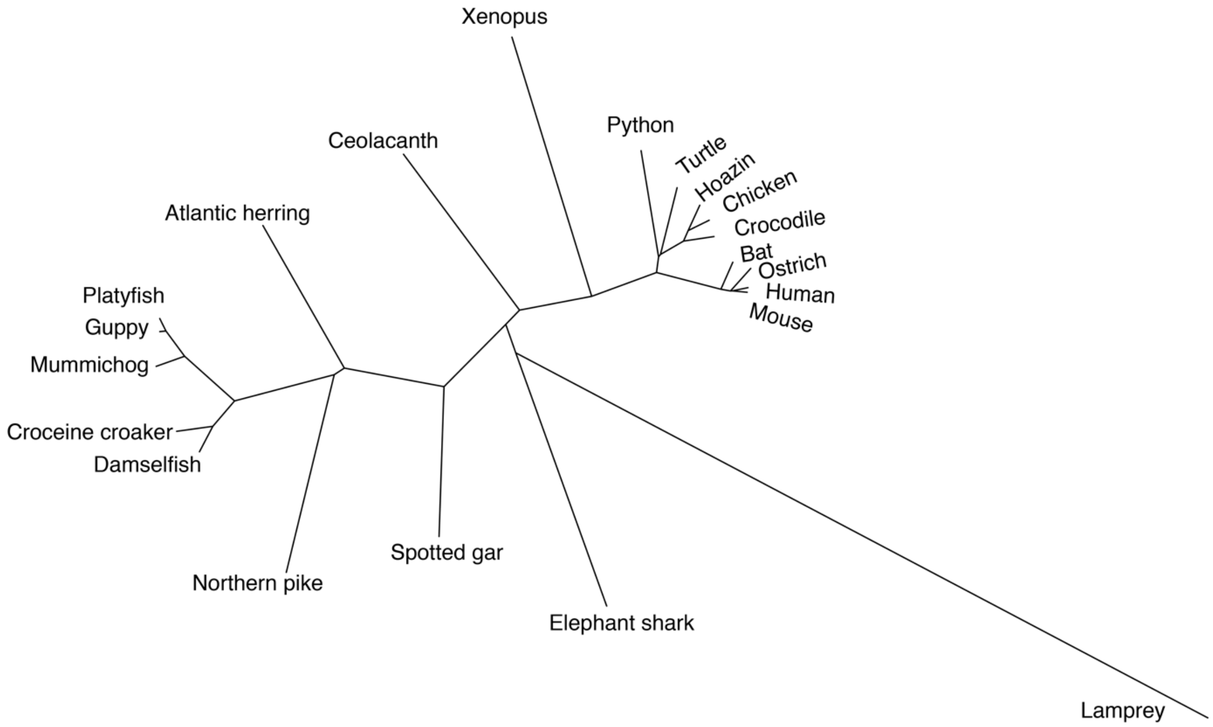

Supplementary Figure 2 | Phylogenetic analysis of ASTN-2 based on sequence alignment. Protein amino acid sequences of ASTN-2 were retrieved from NCBI where the full-length sequences were available. Sequences of ASTN-2 from crocodile, elephant shark and lamprey were assembled from genome shotgun sequences. ASTN-2 from lamprey appears to be the most diverse protein among the vertebrate species above, although only one version of the ASTNs was found in its genome while other species usually contain two (ASTN-1 and ASTN-2), probably indicating that ASTNs were split into two copies early on in vertebrate evolution. Sequence alignment was carried out in Clustal Omega and the phylogenetic tree was generated using FITCH and DRAWTREE as part of PHYLIP package. See main text for references.

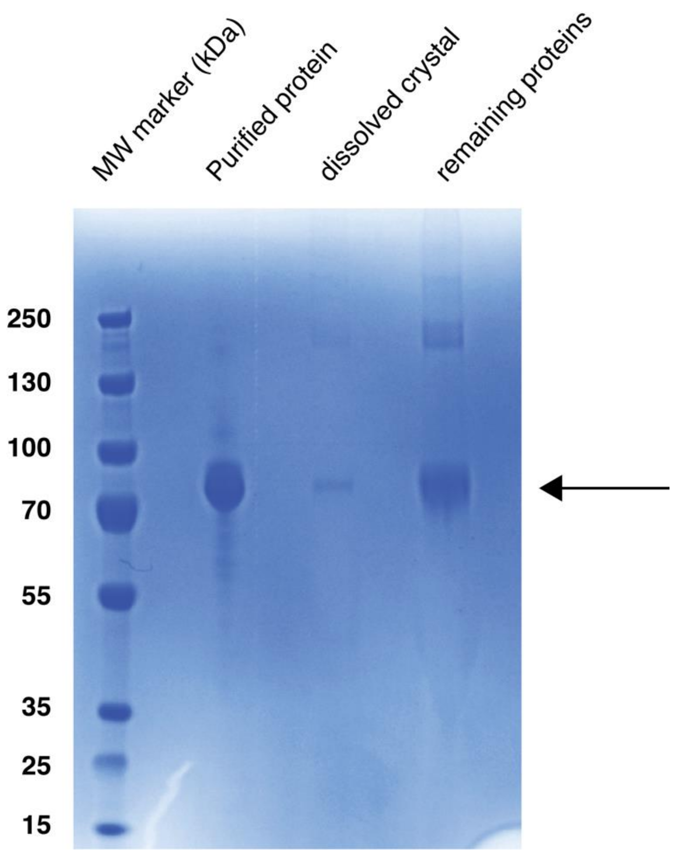

Supplementary Figure 3 | SDS-PAGE analysis of ASTN-2 crystals. A relatively large crystal (20x40x80µm) was dissolved in SDS-PAGE running buffer picked up from the crystallisation drop. Proteins remaining in crystallisation drops collected from 5 wells were prepared in the same way. The molecular weights from purified proteins and dissolved crystals were shown to be the same. The arrow indicates the protein bands with expected size.

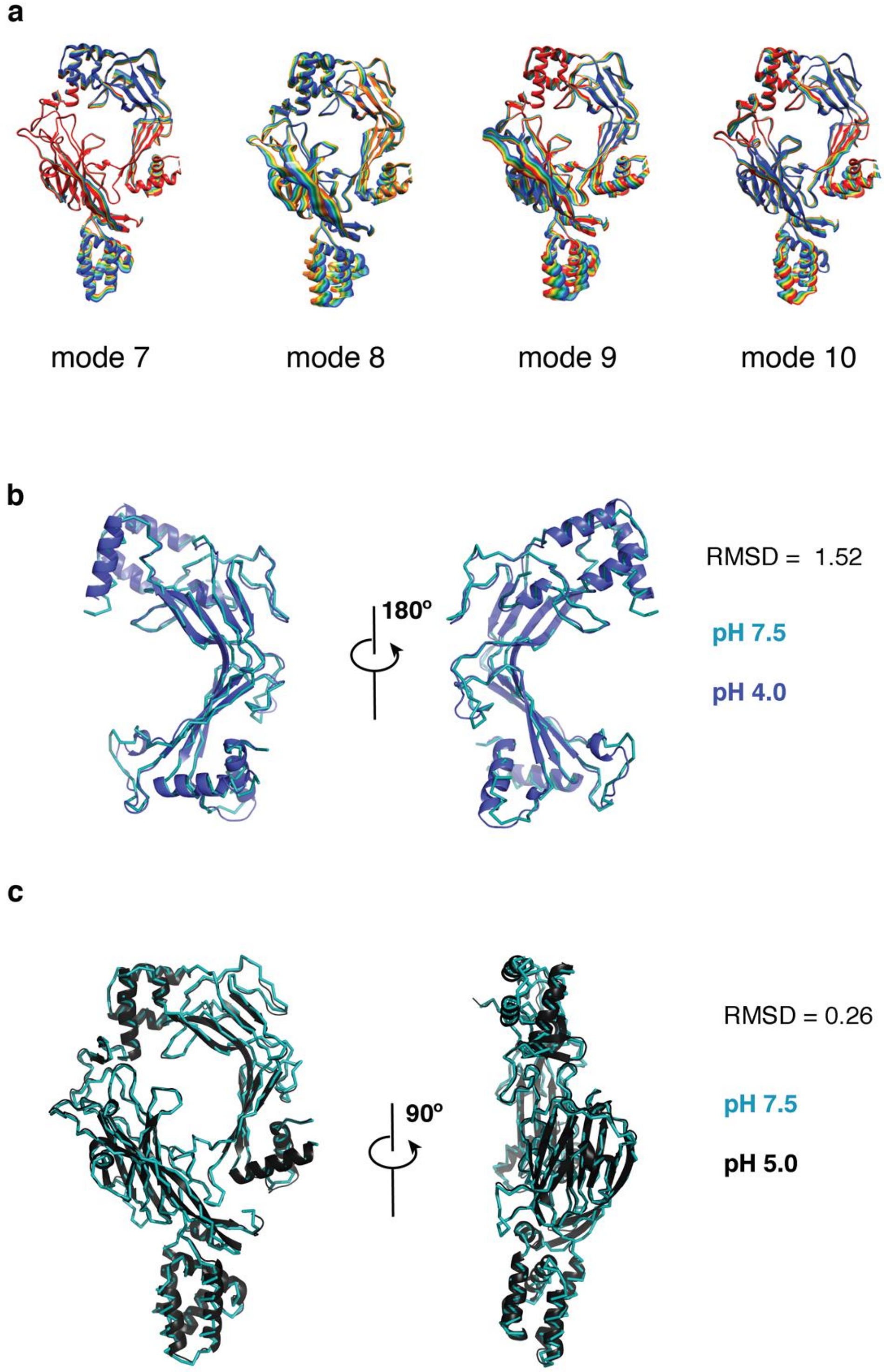

Supplementary Figure 4 | Normal modes analysis of the ASTN-2 endodomain structure and ASTN-2 at pH 5 and 4. (a) Normal modes were computed using the El Nemo webserver; see main text Materials and Methods for details and a reference. The first six modes in a normal modes analysis are trivial, being translational and rotation motions in three dimensions; the first non-trivial mode is therefore normal mode 7 and we show the first five such non-trivial modes indicating the principal regions of flexibility within the endodomain of ASTN-2. (b) ASTN-2 MACPF domain at pH 4 (blue cartoon) in superimposition with itself crystallised at pH 7.5 (cyan ribbon). (c) ASTN-2$_{649\text{-}1288}$ structure at pH 5 (black cartoon) superimposed with itself at pH 7.5 (cyan ribbon). EGF3 domain is not resolved in the structure.

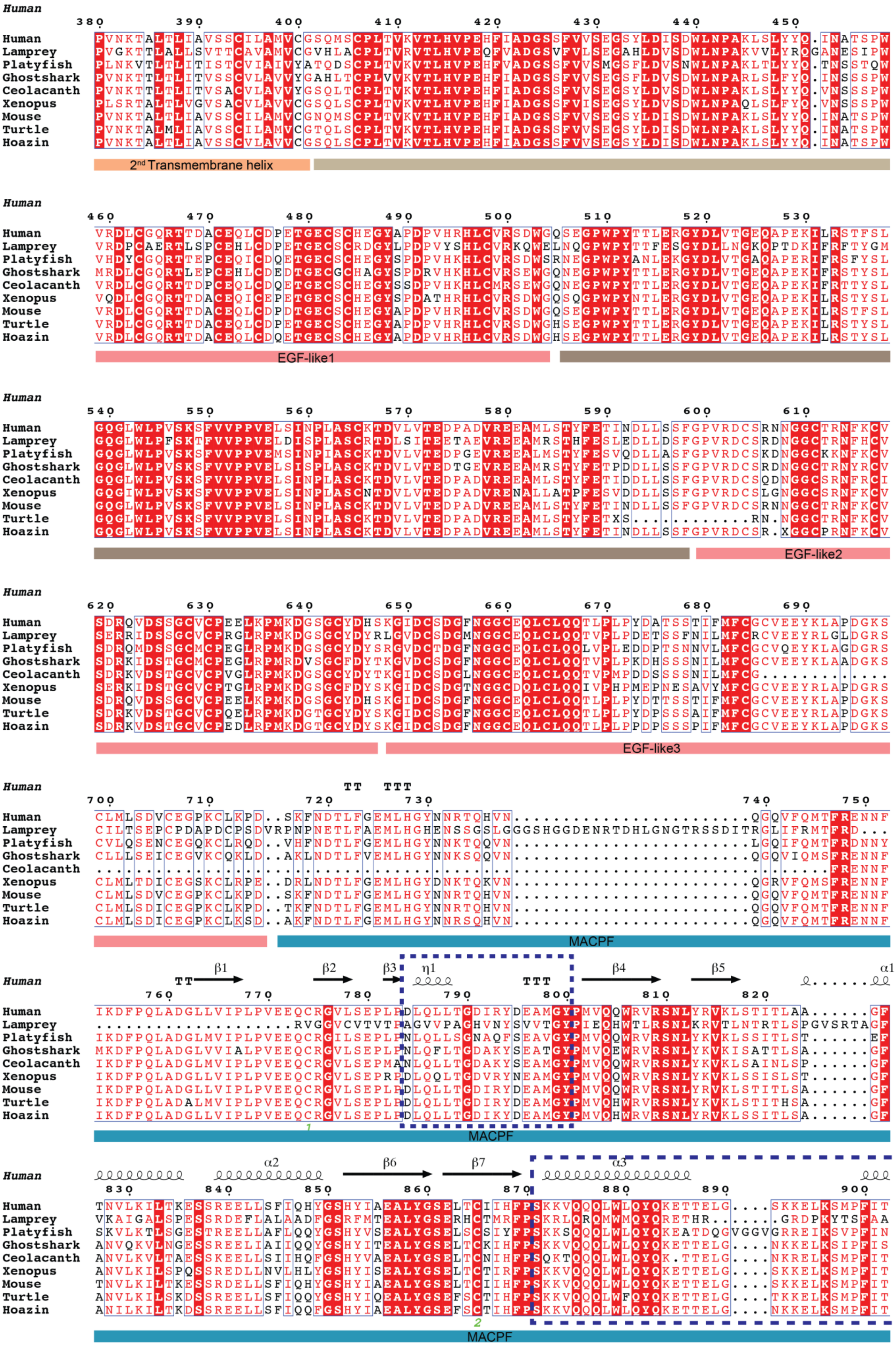
Human
Lamprey
Platyfish
Ghostshark
Ceolacanth
Xenopus
Mouse
Turtle
Hoazin
2nd Transmembrane helix
EGF-like1
EGF-like2
EGF-like3
MACPF
β1
β2
β3
η1
β4
β5
α1
α2
β6
β7
α3
TT
TTT

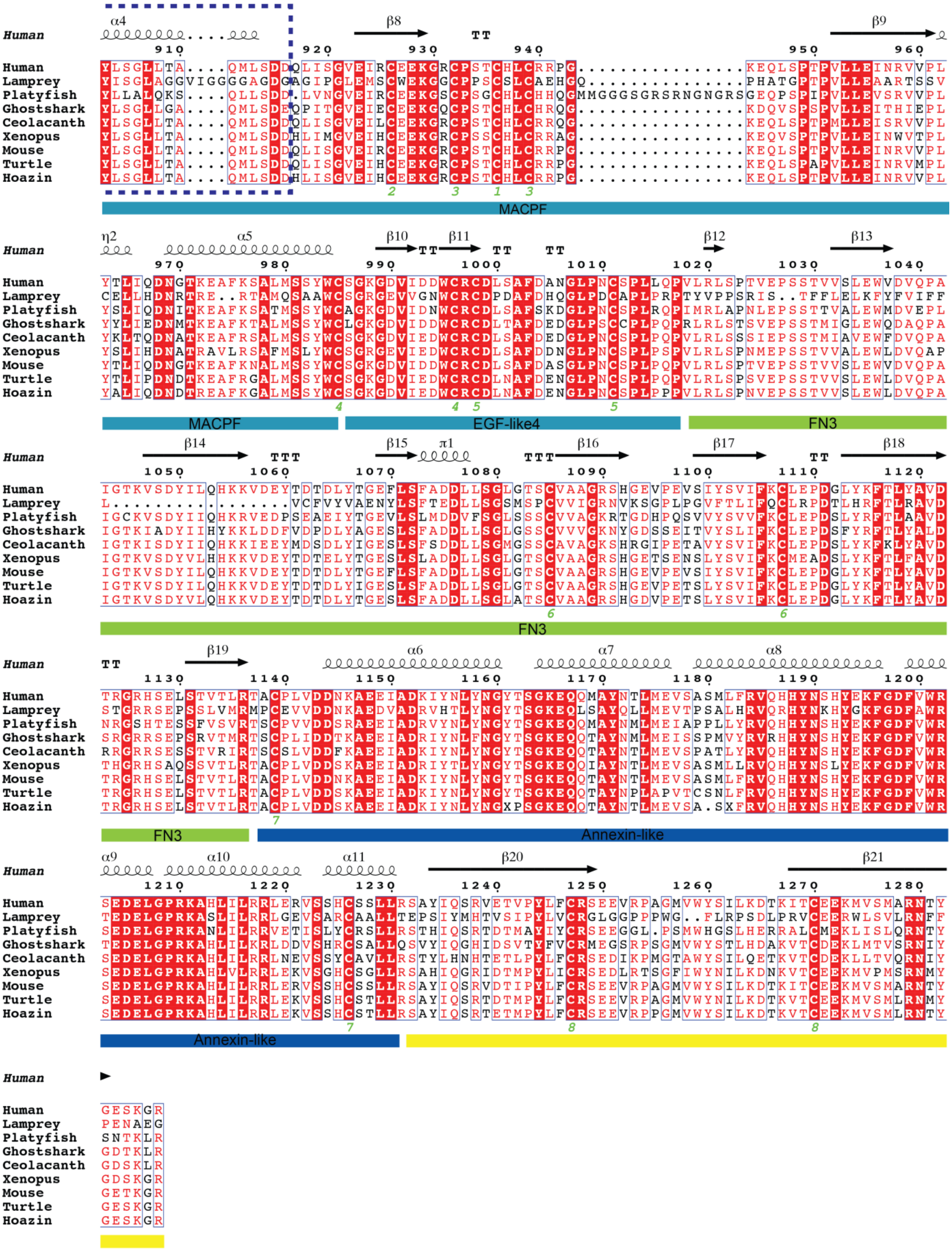

Supplementary Figure 5 | Sequence alignment of ASTN-2 endodomain after the second transmembrane helix. Two loops region of ASTN-2 MACPF domains are highlighted with a dashed-line rectangle. The figure is rendered in ESPript 3 (http://espript.ibcp.fr); see main text for reference.

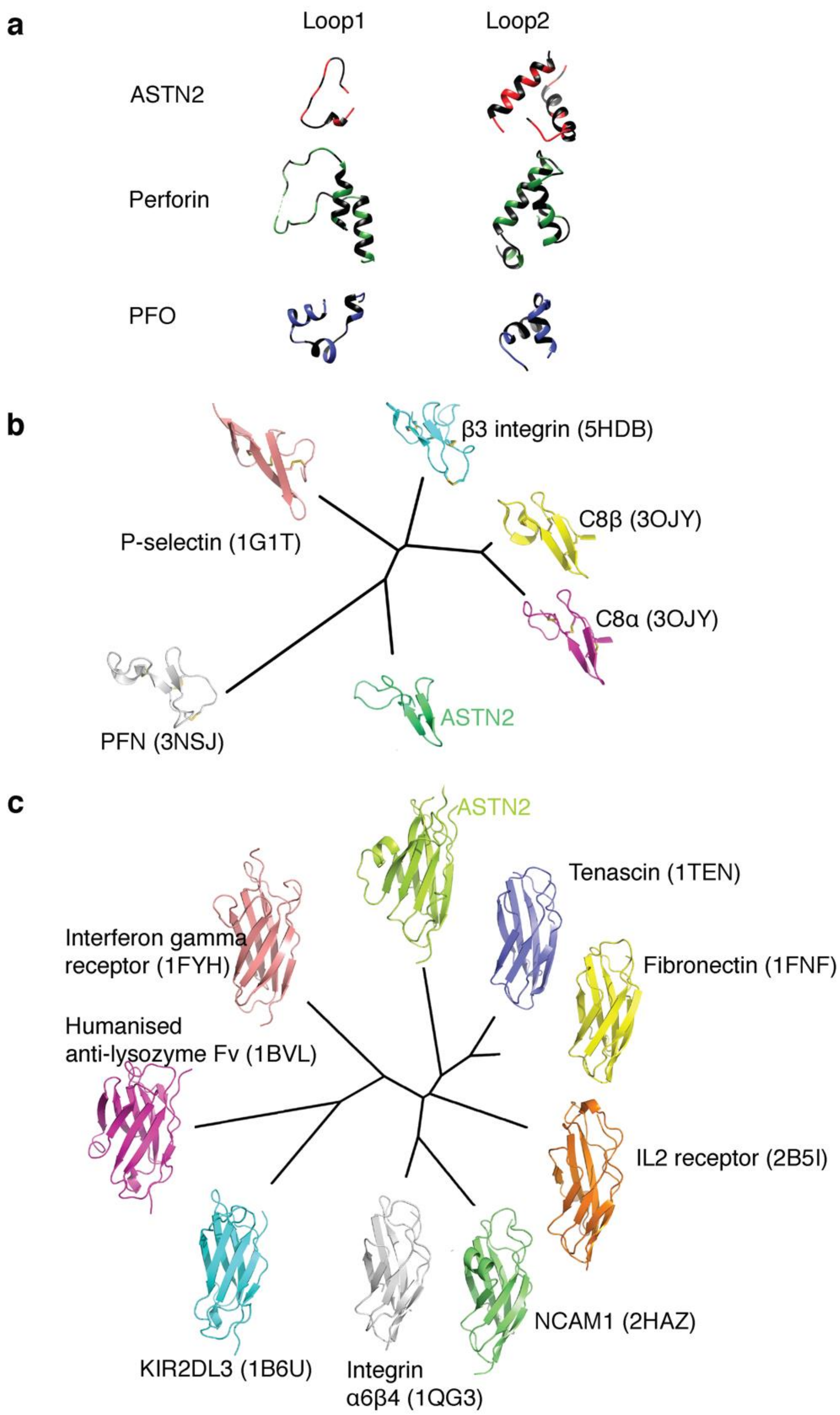

Supplementary Figure 6 | Structural comparison of ASTN2 loops and phylogenetic analysis of EGF-4 and Fn(III). (a) Loop1 and loop2 regions from ASTN-2, perforin-1 and PFO showing the abbreviated loop1 of ASTN-2. **(b)** Phylogenetic analysis of ASTN-2 EGF4 domain. **(c)** Phylogenetic analysis of ASTN-2 Fn(III) domain among other Fn(III) domains. PDB codes are shown in brackets.

a

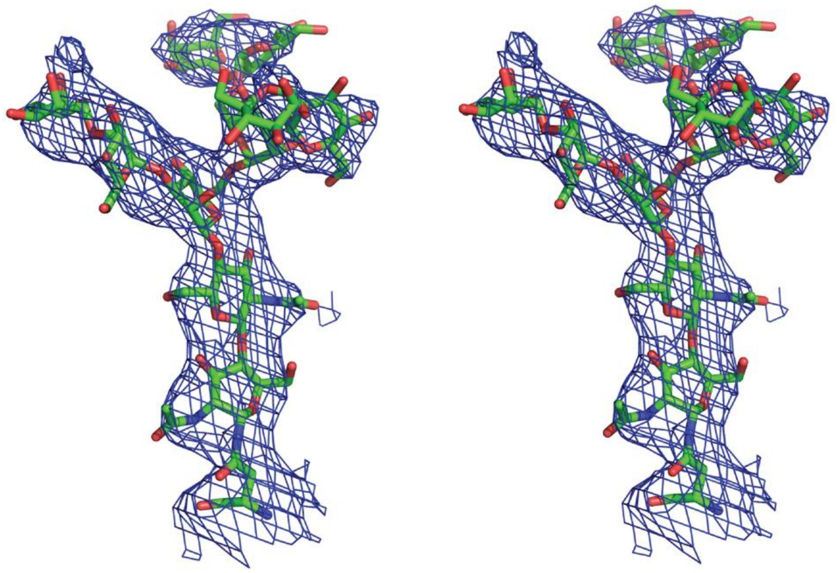

b

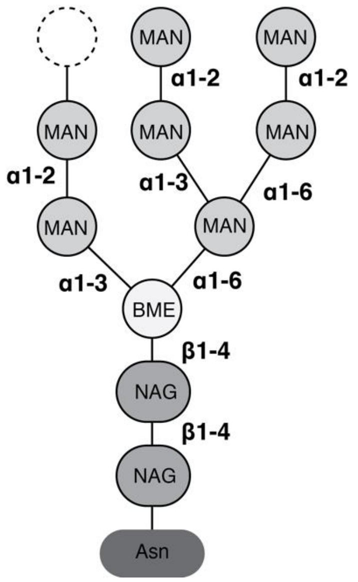

Supplementary Figure 7 | The N-linked glycosylation at N732. (a) Stereo diagram showing the ordered electron density of N-linked glycosylation at N732. The N732 residue is shown in the bottom of the electron density. **(b)** Diagrammatic representation of N-linked glycosylation. There are three branches of oligosaccharides, with 9 mannoses in total. The dashed circle in the cartoon represents the missing (disordered) α-mannose which is not clearly resolved in electron density. MAN: α-mannnose; BME: β-mannose; NAG: N-acetylglucosamine.

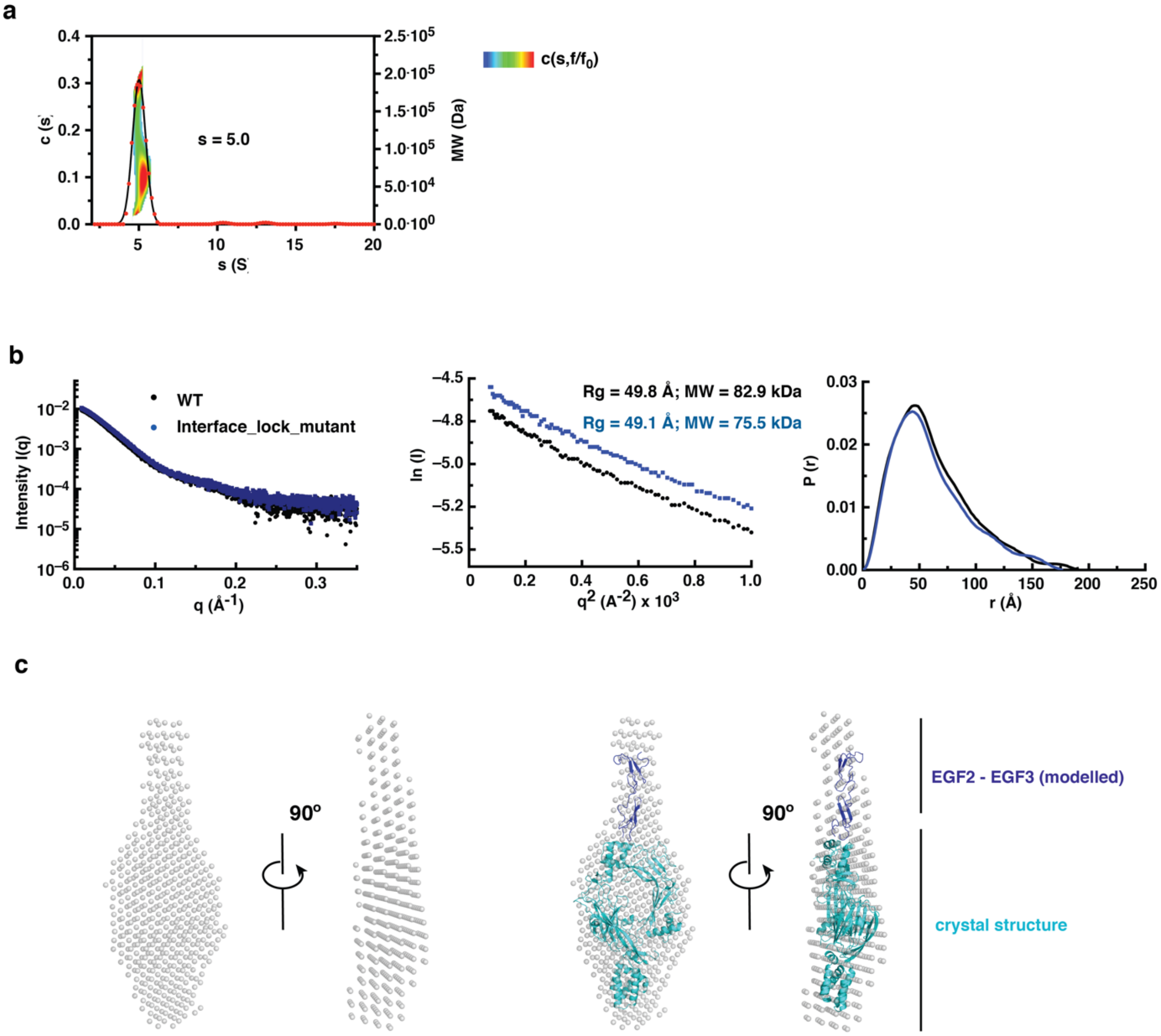

Supplementary Figure 8 | Analytical ultracentrifugation (AUC) and SAXS analysis of ASTN-2. (a) AUC analysis of ASTN-$2_{601\text{-}1288}$ WT showing the monomeric property of the protein in solution, with sedimentation coefficient of 5.0 s. **(b)** SAXS analysis of ASTN-$2_{601\text{-}1288}$ WT and interface locking mutant. The scattering curves of protein samples at the same concentration (1mg/ml) are shown here, with a similar calculated Rg value and distance distribution function. **(c)** *ab initio* model of ASTN-$2_{601\text{-}1288}$ WT calculated from the P(r) function in damfilt. The P(r) function was calculated from merged data including low-resolution data from a high-concentration sample (4.92mg/ml) and high-resolution data from a low-concentration one (1.27mg/ml).

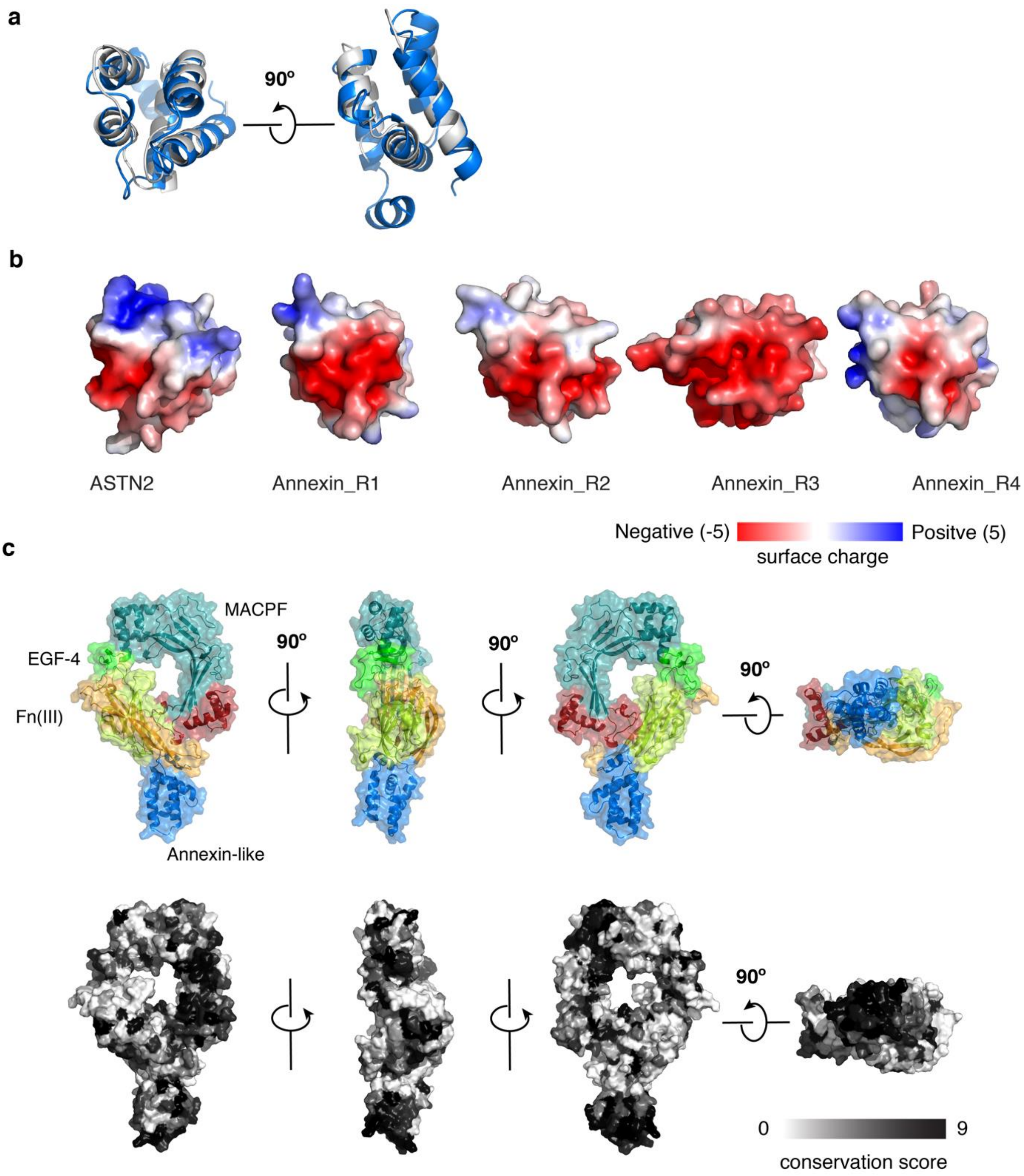

Supplementary Figure 9 | Superimposition of ASTN-2 annexin-like domain and surface charge distribution comparison. (a) ASTN-2 annexin-like domain in superimposition with Annexin V (PDB: 1AVR) repeat 1 domain. **(b)** Comparison of ASTN-2 annexin-like domain surface charge with each repeat from Annexin V; each domain is shown in the same equivalent orientation as the others. The surface electrostatic potential was calculated using APBS (see main text). **(c)** Domain conservation calculated from sequences of ASTN-2 in species the same of that in supplementary Fig. 2 using Consurf server (http://consurf.tau.ac.il) (see main text for reference). The conservation score was plotted from 0 to 9, with colour gradient from white to black.

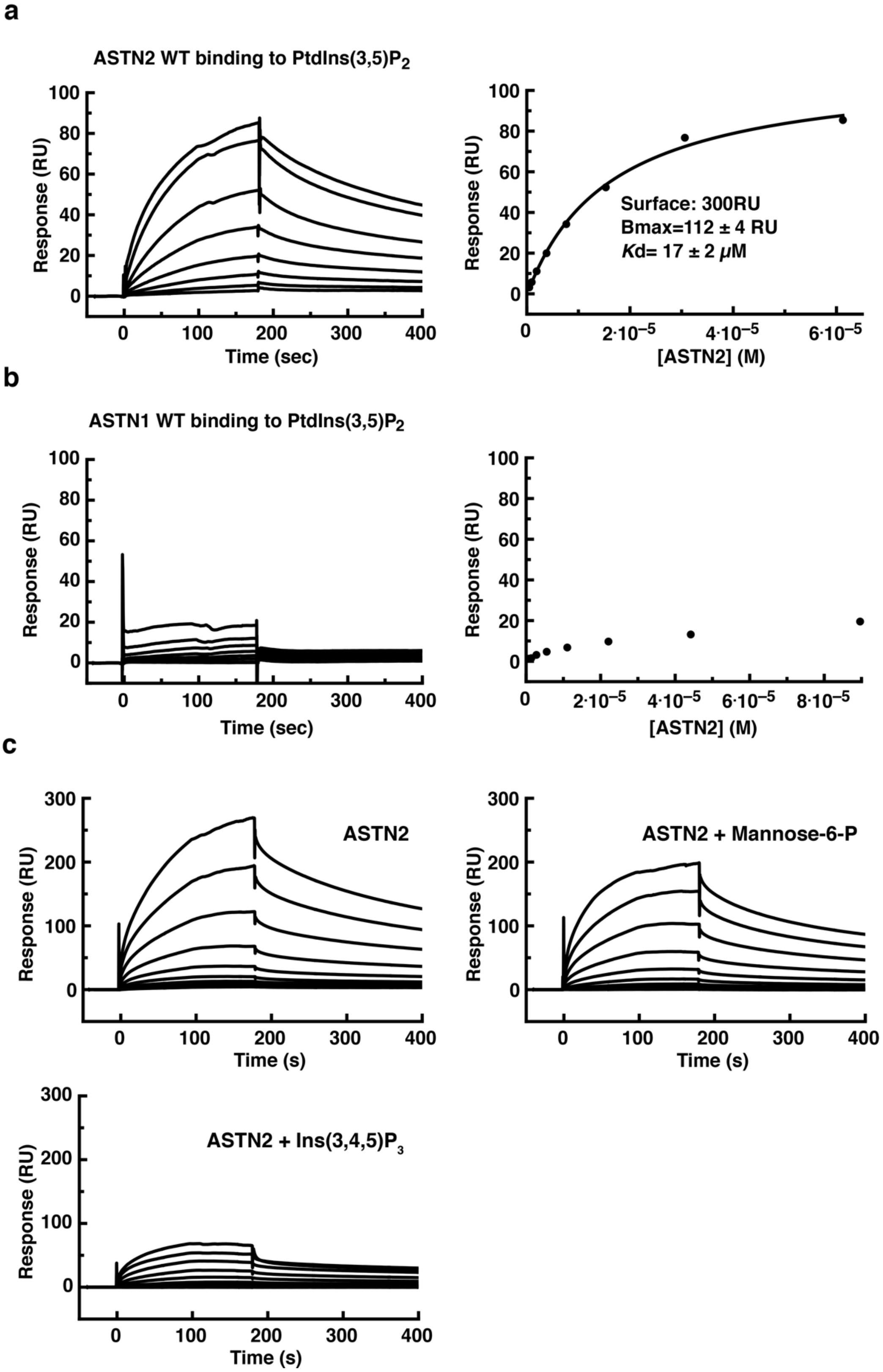

Supplementary Figure 10 | SPR experiments of ASTN-1 and ASTN-2 interactions with inositol phosphate species. (a) SPR experiment showing interaction of PtdIns(3,5)P_2 with ASTN-2 (residues 601 - 1288); left: SPR sensogram, right: fitted data. **(b)** SPR study of PtdIns(3,5)P_2 with ASTN-1. The ASTN-1 construct here was composed of the equivalent regions to the ASTN-2 construct. **(c)** Uses ASTN-2 (residues 701 – 1288, i.e. lacking the EGF2-EGF3 tandem). In this SPR competition assay we show first ASTN-$2_{701\text{-}1288}$ alone binds Ins(3,4,5)P_3 headgroups equivalently to ASTN-$2_{601\text{-}1288}$. We then show that Ins(3,4,5)P_3 competes with the immobilized PtdIns(3,4,5)P_3 binding while mannose-6-phosphate does not.
